# When local isn't best

**DOI:** 10.1111/eva.12090

**Published:** 2013-08-14

**Authors:** Thomas A Jones

**Affiliations:** USDA-Agricultural Research Service, Forage and Range Research LaboratoryLogan, UT, USA

**Keywords:** ecological restoration, local adaptation, local is best, native plants, novel ecosystems, plant materials, provenance

## Abstract

This paper attempts to explain circumstances under which local may be or may not be best. Natural selection may lead to local adaptation (LA), or it may be constrained by gene flow, founder effects, small population size, genetic drift, and archetype. ‘Specialist’ species display greater LA than ‘generalist’ species. Local genotypes are to a certain extent transient, being a consequence of past historical genetic patterns. Two recent meta-analyses found that while local performance exceeded the performance of a randomly chosen nonlocal population in 71% of comparisons, general adaptation across environments was as frequent as LA. Genotypes for restoration are most likely to be effective if they are adapted to current site conditions. As environmental change accelerates, both globally and locally, exceptions to ‘local is best’ may increase. For these reasons, ‘local is best’ may be better thought of as a testable hypothesis rather than as a general assumption. While either local or nonlocal plant material may be most effective for restoration practice depending on individual circumstances, local material will continue to be the first choice for restoration practitioners whenever this option is feasible and effective.

## Introduction

For the practice of ecological restoration, the plant materials of choice are often local in origin (Namkoong 1969; Brown and Amacher 1999; Wilkinson 2001; Rogers and Montalvo 2004; Broadhurst et al. 2008; Brown et al. 2008). While supporting this preference for local material, that is, the primary restoration gene pool, I have also acknowledged that nonlocal material may be a viable option when local material is unavailable, prohibitively expensive, or no longer adapted due to ecosystem change (Jones 2003). The ‘local-is-best’ (LIB) assumption is often invoked to justify the choice of local plant material, and its use is supported by many data sets, as summarized by Johnson et al. (2010). Parameters used to determine what is ‘best’ vary, but they are typically components of fitness (Leimu and Fischer 2008; Hereford 2009) or traits relating to aboveground plant size or biomass (Leimu and Fischer 2008). Fitness parameters, as discussed by Kawecki and Ebert (2004), are preferred to assess the importance of local adaptation (LA), but they are difficult to measure in the field (Kassen 2002).

Many cogent arguments support the LIB plant material paradigm. For example, Endler et al. (2010) detailed several dangers regarding nonlocal material. These include the disruption of genotype frequencies across geographic space, the introduction of genes poorly adapted to local conditions, inbreeding and domestication resulting from artificial selection, the fixation of maladapted genotypes due to genetic drift, the disruption of eco-adaptation (i.e., local patterns of gene interaction among species), an impaired ability to adapt to future environmental change, and resultant cascade effects at the community level. Nearly a decade earlier, Sackville Hamilton (2001) argued that biodiversity conservation is the primary reason to control provenance and that while introduction of nonlocal germplasm may augment genetic variation, such variation is inappropriate. Montalvo et al. (1997) noted the importance of ‘genetic memory’, the genetic legacy resulting from a history of natural selection, in local material. These authors also noted the dangers of ‘swamping’ local genes and outbreeding depression that may result from the introduction of nonlocal material. In addition, local plant material may be preferred based on the precautionary principle of ecological restoration (Smith et al. 2009).

While these arguments have considerable merit, they may be less applicable for seriously degraded lands. The best management practices for sustaining mostly pristine lands may differ from those for restoring novel ecosystems (Hobbs et al. 2006). This is particularly noteworthy because such ecosystems are expanding at the expense of pristine landscapes worldwide (Ellis et al. 2012). Consequently, it is likely that ecological restoration will be increasingly directed toward ameliorating highly degraded lands of the sort that Aldo Leopold labored to restore (Aber and Jordan 1985). Leopold, a professor of wildlife management at the University of Wisconsin, is regarded by many as the father of ecological restoration in North America. In 1933, he bought an abandoned farm along the Sauk River in Wisconsin, and he worked to restore it until his death while fighting a neighbor's wildfire 15 years later (Callicott 1987).

The importance of LA in plants is widely recognized (Linhart and Grant 1996; Johnson et al. 2010), and the mechanisms by which it operates are largely understood (Kawecki and Ebert 2004). However, reports of exceptions to the LIB paradigm are increasing (e.g., Leimu and Fischer 2008; Hereford 2009; Smith et al. 2009; vander Mijnsbrugge et al. 2010; Carter and Blair 2012). Empirical results of local versus nonlocal comparisons will likely continue to be equivocal, and it is unlikely that there is one ‘best’ approach. Arguments for using local plant materials are both valid and convincing, but considerations that favor a role for nonlocal materials in ecological restoration may be less familiar. The purpose of this paper is not to assert that either local or nonlocal genotypes are generally superior, but rather to simply suggest that circumstances exist under which the use of nonlocal genotypes may be a viable restoration option. Here, I highlight 10 issues (Table 1) that describe these circumstances in hopes of stimulating a discussion of potential situations for which local plant materials may be or may not be best.

**Table 1:** Ten issues to be considered when choosing among local and nonlocal populations for ecological restoration

Ecological issues
1. The ecological state and the choice of a restoration target
2. Novel ecosystems and anthromes
3. Ecosystem patterns, processes, and functions
Genetic issues
4. Variation in genetic diversity among populations of a species
5. Outbreeding depression and genetic diversity
Evolutionary issues
6. The role of natural selection
7. Local adaptation and general adaptation
8. Co-evolution and ecological fitting
Issues relating to restoration practice
9. Testing methodology
10. The relative frequency of opportunity for nature versus restoration practice

## Ecological issues

### The ecological state and the choice of a restoration target

The use of a historic state as a restoration target is recommended when feasible (Jackson and Hobbs 2009). However, when an ecosystem state change has occurred or future environmental conditions are expected to move outside the historic range of variability (Szabό 2010), such an approach may be less tractable (Choi 2004, 2007; Jackson and Hobbs 2009). Under such circumstances, Kessler and Thomas (2006) suggested managing for ecosystem resilience. Because changing climates may alter population dynamics, geographic ranges, and community and ecosystem structure and function, it is conceivable that recent immigrants may be pre-adapted by chance and even fare better than longer-term residents (Walther et al. 2009). In fact, cryptogenic species, those with an uncertain status as being either native or introduced and subsequently naturalized, are remarkably frequent (Carlton 1996).

For rangelands in the Intermountain West USA, state-and-transition models have displaced steady-state successional models (Stringham et al. 2001). Therefore, the restoration goal is transition from one stable state (a degraded system) to another (the desired restoration target) by crossing a threshold (Temperton et al. 2004). Such thresholds commonly feature hysteresis, whereby the trajectory from state A to state B differs from the return trajectory (Beisner et al. 2003; Bestelmeyer et al. 2011). While the transition from a desirable state to an undesirable state may be relatively easy, efforts to restore an undesirable (and often recalcitrant) state may be more difficult (Beisner et al. 2003). Genotypes that effectively restore desired ecological processes and ecosystem function must necessarily be adapted to the modified conditions and may not always be local in origin.

### Novel ecosystems and anthromes

Novel ecosystems are those which have newly arisen through the intentional or inadvertent activities of humans, yet are self-perpetuating (Hobbs et al. 2006). Anthromes are human-influenced biomes that are now pervasive and surround Earth's remaining natural biomes (Ellis and Ramankutty 2008). Lands are subject to perturbations, alternative states, and at-risk community phases (Bestelmeyer et al. 2010). It is these, rather than pristine lands, which are the most frequent restoration targets, and consequences of this global human footprint can best be addressed by ecological restoration in the tradition of Aldo Leopold (Schmitz 2012). An argument for the exclusive use of local plant materials is that nonlocal materials are not sustainable (Montalvo et al. 1997), yet an analogous argument can be made regarding local materials when used in novel ecosystems (Jones and Monaco 2009). Broadhurst et al. (2008) recognized that novel ecosystems may require stress-tolerant genotypes and perhaps nonlocal, although functionally similar, species. However, the recognition of novel ecosystems and anthromes should never be used to minimize the value of pristine environments, to contend that novelty is desirable, to adopt a *laissez-faire* attitude toward traditional conservation, to justify the abandonment of efforts to control invasive species, or to display professional hubris (Hobbs et al. 2013; Standish et al. 2013).

### Ecosystem patterns, processes, and functions

Contemporary naturally occurring genetic patterns are simply the result of environmental forces driving natural selection on previous historic genetic patterns (Sgrò et al. 2011). Because both selective forces and ambient genetic patterns are subject to change, current patterns are labile rather than fixed entities (Sgrò et al. 2011). Consequently, while natural genotypes may have inherent value, it may be unrealistic to expect them to be the best adapted following modification of environmental conditions.

Patterns of local genotypes have been widely regarded as central to the conservation of biodiversity (Sackville Hamilton 2001). Yet an emphasis on patterns can come at the expense of the processes that drive patterns at the landscape level (Wilkinson 2004). Thus, to achieve a desirable restoration outcome in a modified environment, an emphasis on functional plant traits (Pywell et al. 2003; Funk et al. 2008; Jung et al. 2010; Roberts et al. 2010; Clark et al. 2012) that can redirect ecological processes to overcome restoration obstacles, for example priority effects, plant interference, and undesirable positive feedbacks (Jones et al. 2010), may be considered in addition to an emphasis on taxonomic and genetic patterns (McGill et al. 2006). Choi (2007) called for rehabilitating ecosystem function to stimulate the assembly of ecosystems for a sustainable future.

## Genetic issues

### Variation in genetic diversity among populations of a species

Genetic variation may be greater in certain portions of a species' distribution than others. Genetic diversity may be expected to be low due to a founder effect at the extremity of a species' distribution (Davis and Shaw 2001) or when natural populations have become fragmented, resulting in small population sizes and consequent genetic drift (Ashley et al. 2003; Broadhurst et al. 2008; Weeks et al. 2011). For outcrossing species that display inbreeding depression, rather than adhering to a strictly local protocol that may reflect the low genetic diversity common in small fragmented populations, adaptive potential may be better achieved with high-quality and genetically diverse seed (Broadhurst et al. 2008; Breed et al. 2013). However, in their meta-analysis, Leimu and Fischer (2008) found that small populations were less likely to display LA and more likely to display local maladaptation regardless of cross- versus self-pollinating mating system. Likewise, Hereford (2010) found no difference between mating systems for the development of LA. Kramer and Havens (2009) suggested that human-mediated gene flow may counteract genetic erosion by restoring adaptive genetic variation.

Davis and Shaw (2001) explained that genetic variation is often greater near the center of a species' distribution because of the proximity of various populations that possess adaptation to a variety of environments. Plant material development in bluebunch wheatgrass (*Pseudoroegneria spicata* [Pursh] A. Löve) has capitalized on this concept, as all released plant materials of this species originate in southeastern Washington, USA, a region where several metapopulations converge (Larson et al. 2004). The P-7 bluebunch wheatgrass germplasm, a multiple-origin polycross of 25 local populations, was designed to capture much of the genetic variation in this region in order to provide augmented genetic variation upon which natural selection may act (Larson et al. 2000).

A geographic hotspot of genetic diversity may be useful for developing restoration plant material for another region, particularly if it occupies a similar environment to that of the restoration target site, a phenomenon referred to as ‘recurrence’ (Johnson et al. 2004). In the northern hemisphere, distant glacial refugia, either southerly or coastal, or dry-climate refugia, on hilltops (Pielou 1991), may still contain relict genetic material of populations that retreated from changing climates in the geologic past. Likewise, less-distant-scattered microrefugia, presumably characterized by sheltered topography and favorable microclimate (Rull 2009), could also provide such material.

### Outbreeding depression and genetic diversity

A commonly stated caution regarding the use of plant materials that are either nonlocal or augmented for genetic diversity is the danger of outbreeding depression (Fenster and Galloway 2000; Keller et al. 2000; Hufford and Mazer 2003; Crémieux et al. 2010; Laikre et al. 2010). Using a mathematical approach, however, Frankham et al. (2011) concluded that outbreeding depression is most likely when parents are distinct species, display fixed chromosomal differences, have not exchanged genes in the last 500 years, or occupy distinct environments. They argued that excessive concerns about outbreeding depression may result in a failure to address the greater problem of inbreeding depression. Strategic mixing of plant populations to deliver enhanced genetic variation and evolutionary resilience has been widely advocated in recent years (McKay et al. 2005; Broadhurst et al. 2008; Jones and Monaco 2009; Sgrò et al. 2011; Weeks et al. 2011). However, a belief in the merit of local plant material and an emphasis on outbreeding depression have discouraged the use of mixed-source populations despite logistical and economic advantages (McKay et al. 2005; Weeks et al. 2011). vander Mijnsbrugge et al. (2010) emphasized precaution, recommending the mixing of populations only when hard evidence of inbreeding depression is present or when populations are very small.

It is important to strike a balance between prevention of inbreeding depression and outbreeding depression (McKay et al. 2005). However, this may be complicated by the masking of outbreeding depression by inbreeding depression (Edmands 2007). In case of nonthreatened allogamous species, the repercussions of inbreeding depression may be more serious, as outbreeding depression resulting from local × nonlocal hybridization may be corrected by natural selection over the course of several generations (Carney et al. 2000; Erickson and Fenster 2006). While these ‘fused’ populations may exhibit fitness advantages (Weeks et al. 2011) or display adaptation to an expanded ecological range (Rieseberg et al. 2003), Endler et al. (2010) warned that maladapted genotypes may become fixed by genetic drift before natural selection can facilitate fitness rebound.

## Evolutionary issues

### The role of natural selection

Local plant materials are sometimes portrayed to be an ideal, and nature and natural selection, to be inviolate. Stephen Jay Gould regarded the belief that natural selection is an optimizing process as a misinterpretation of evolution, yet one that is pervasive in modern culture (Gould and Lewontin 1979; Gould 1998). Instead, Gould (1998) asserted that evolution operates in a matrix of random effects, such as chaos and contingency, and its products are simply a result of sorting among locally available genotypes following the evolutionary path of least resistance (Stebbins 1970). Limits on the optimality of the evolutionary process may be imposed by founder effects, linkage disequilibrium, allometry, pleiotropy, the archetype (the genetic prototype, i.e., the genetic material available at a site that fuels the evolutionary process), and compensatory trade-offs among traits (Harper 1982). LA may also be hindered by gene flow, genetic drift, environmental fluctuations over time, and insufficient genetic variation (Kawecki and Ebert 2004).

Because these forces may confound natural selection, an optimal pattern of LA does not necessarily result from evolution (Kawecki and Ebert 2004). The absence of the most adapted genotype locally may relate to random historical events or geographic constraints. For example, native species often compare poorly with introduced species never before exposed to the native environment (Goodenough 2010), and many immigrant species are exceptionally well adapted to their new environments as a consequence of traits that evolved elsewhere (Mack 2003). Mack (2003) made the obvious point that a community can only be composed of species that have arrived at its location. Likewise, selection for LA cannot operate on material absent from the site, despite the fact that it may be better adapted than local material (Harper 1982; White and Walker 1997; Gould 1998).

In some cases, nonlocal genotypes of local species have become invasive following introduction. For example, cryptic invasions by non-native genotypes of common reed (*Phragmites australis* [Cav.] Trin. ex Steudel) have expanded this species' North American range and displaced native genotypes (Saltonstall 2002). In addition, the introduction of smooth cordgrass (*Spartina alterniflora* Loisel.) into San Francisco Bay and its subsequent hybridization with and introgression into a genetically similar native, California cordgrass (*Spartina foliosa* Trin.), have resulted in highly fertile and strongly invasive transgressive segregates (Ayres et al. 2008). The conversion of unvegetated tidal flats into *Spartina* meadows has effected a change from an algal-based to a detritus-based food web, resulting in reduced trophic support for fish and migratory birds (Levin et al. 2006). Bischoff et al. (2010) concluded that while the use of local provenance does not always guarantee superior performance, it does avoid the invasive spread of alien genotypes.

One might expect a lower likelihood of a nonlocal genotype of a native species being invasive than for an introduced species based on the greater genetic similarity of the former to the native genotype. In the Intermountain West, USA, invasive genotypes of native species have not been a problem to date. In fact, it has been difficult to find genotypes of native species that are well adapted to the region's modified soil and fire regimes and able to establish on lands invaded by exotic annual grasses (Jones and Monaco 2009). Nevertheless, a protocol is in place to assess the potential for invasiveness prior to plant material release (Jones and Robins 2011, section 3.4).

### Local adaptation and general adaptation

Kawecki and Ebert (2004) stated that environmental heterogeneity favors natural selection for adaptive phenotypic plasticity, but if this does not occur, selection for LA may result if a genotype × environment interaction, in particular antagonistic pleiotropy, for fitness is present. In antagonistic pleiotropy, alleles have opposite effects on fitness in different habitats, no single genotype is superior in all habitats, and trade-offs in adaptation among habitats result (Kawecki and Ebert 2004). Temporal heterogeneity favors natural selection for generalist genotypes, while heterogeneity across sites favors selection for LA, assuming restricted gene flow (Kassen 2002). Consequently, for divergent selection to generate LA, environmental variation must be substantially greater across space than across time (Kawecki and Ebert 2004; McKay et al. 2005).

Kawecki and Ebert (2004) recommended the use of the ‘local versus foreign’ comparison to test for the presence of LA. ‘Local versus foreign’ comparison involves reciprocal testing of many pairs of populations at both the local and foreign sites, meaning that populations originating at sites A and B are compared at both sites A and B. If the local population is superior at both sites, LA may be presumed. However, if population A exceeds population B at both sites, the evidence is for general adaptation (GA) in favor of population A. The frequency of population A being superior to population B at site A is an upwardly biased estimate of LA, as LA also requires population A to be inferior to population B at site B (Hereford 2009).

Two recent meta-analyses (Leimu and Fischer 2008; Hereford 2009) have reported that instead of LA being far more important than GA, as is often presumed (McKay et al. 2005), frequencies of GA and LA are similar (Table 2). Hereford (2009), who considered only fitness-related traits, reported a slightly higher frequency of LA and local maladaptation than Leimu and Fischer (2008), who considered both fitness-related and biomass-related traits. Both meta-analyses reported a 71% frequency of local superiority in one-on-one comparisons, so more often than not, local is probably superior to a randomly selected nonlocal population. In the absence of information regarding the performance of nonlocal commercially available populations relative to the local population in environments similar to the restoration site (perhaps the most common occurrence in restoration practice), the local population may be preferred. To this end, Bower et al. (2011) proposed provisional seed zones based on average maximum temperature and annual precipitation for herbaceous species.

**Table 2:** Conditions necessary to declare local adaptation (LA), general adaptation (GA), or local maladaptation (as defined here) and their frequencies in one-on-one comparisons of populations A and B (local versus nonlocal) at sites A and B in two meta-analysis studies. Population A originates at site A, and population B originates at site B. Note that GA may occur in either of two ways (population A is generally superior across sites A and B, or population B is generally superior across sites A and B). The terminology and associated conditions employed here differ from the terminologies and conditions of both meta-analysis papers, which also differ from one another (see footnotes)

	Superior population			
			
	At site A	At site B	Leimu and Fischer (2008)	Hereford (2009)
LA*	A	B	45%	48%
GA†	A	A	51%	43%
GA	B	B		
Local maladaptation‡	B	A	3%	9%
Number of one-on-one comparisons (*n*)			1032	892
Local superior to nonlocal			71%	71%

* Referred to as ‘POS-POS’ by Leimu and Fischer (2008) or ‘fitness trade-off’ by Hereford (2009).

† Referred to as ‘NEG-POS’ or ‘POS-NEG’ by Leimu and Fischer (2008) or ‘no trade-off’ by Hereford (2009).

‡ Referred to as ‘maladaptation’ or ‘NEG-NEG’ by Leimu and Fischer (2008) or ‘inverse trade-off’ by Hereford (2009).

The 71% estimate may be biased in either direction. For example, the one-on-one meta-analysis comparisons measured whether local is better rather than LIB (Jones 2013). In addition, the comparisons did not involve research-based plant materials, which have been released on the basis of performance testing (Jones 2013). Another factor not considered is the standard restoration practice of matching the nonlocal site of a restoration population to the restoration site on the basis of ecological site similarity (Falk et al. 2001). On the other hand, LA may be undetected in short-term experiments, especially in species that are slow-growing or long-lived (Bennington et al. 2012). Such experiments may exclude critical, although occasional, environmental extremes, omit important segments of the plant life cycle, or miss the scale at which LA is acting (Bischoff and Trémulot 2011). In addition, herbivorous insects may swamp LA of plants with LA of their own and account for a failure to find plant LA (Bischoff and Trémulot 2011). When LA is not detected, Hereford (2009) stated that researchers should test for the mechanisms responsible for its absence.

The authors of the two meta-analyses formulated and tested several specific hypotheses. Leimu and Fischer (2008) failed to reject hypotheses that LA is independent of plant life history, geographic distance between populations, or either spatial or temporal habitat heterogeneity. However, they rejected the hypothesis of independence of LA and population size, finding that large populations (>1000 individuals) were more likely to exhibit LA and suggesting that small populations lacked the genetic potential to develop LA. Hereford (2010) was unable to reject the hypothesis that plant mating system was independent of LA. He also reported a low positive correlation (*r*^2^ = 0.04) between LA and environmental distance among populations, while no relationship was found between LA and phenotypic distance (Hereford 2009). In addition, Hereford (2009) found a very low negative correlation (*r*^2^ = 0.02) between fitness in native and alternate environments. This means that adaptation to one environment is not necessarily accompanied by a loss of adaptation to another, suggesting that any ‘cost of adaptation’ may be minimal. Given such a scenario, development of GA to multiple environments may be possible (Hereford 2009).

### Co-evolution and ecological fitting

The local flora is often valued because it has evolved *in situ*, hence the strong desire for it to be retained intact (Brown and Amacher 1999; Rogers and Montalvo 2004; Ricciardi and Simberloff 2008). The use of nonlocal material may potentially lead to a cascade of negative effects within the ecological community (Sackville Hamilton 2001; Endler et al. 2010). Empirical studies have shown that the relationship between local versus nonlocal plant population origin and insect herbivory is variable (Abdala-Roberts and Marquis 2007; Crémieux et al. 2008; Arany et al. 2009; Ortegόn-Campos et al. 2009). Carter and Blair (2013) found no effect of local versus nonlocal plant population on combined productivity, species richness, or plant community structure of prairie grasslands in the Midwestern USA.

As novel ecosystems are becoming more prominent on Earth, historically authentic, co-evolved biotic assemblages are becoming increasingly scarce (Seastedt et al. 2008). Examples can be cited, both natural (Janzen 1985) and artificial (Wilkinson 2004; Seastedt et al. 2008), in which ecosystems with desirable function have assembled from a flora whose members are neither local nor historically proximal and thus presumably did not co-evolve. Janzen (1985) coined the term ‘ecological fitting’ to describe the complex biotic interactions that may occur in spite of historical allopatry, therefore not deriving benefit from co-evolution. Working in the Santa Rosa National Park in Costa Rica, he witnessed biological interactions that were a result of many species being present together despite having evolved separately in different places and at various times. In what he called ‘the parable of Green Mountain’, Wilkinson (2004) described a highly functional cloud forest on Ascension Island in the south Atlantic made up of species with no history of co-evolution, having been introduced from various continents. Thus, plants do not necessarily need to have co-evolved together either locally or nonlocally to display desirable community-level interactions.

## Issues relating to restoration practice

### Testing methodology

Discussions of LA often begin with references to Clausen et al.'s (1941) research in California that evaluated performance of ecotypes of many species across three sites, ranging from 30 m (Stanford) to 1400 m (Mather) to 3050 m (Timberline). These authors themselves refer to ‘major ecologic races fitted to occupy very contrasting environments’ (p. 243). This work is often cited as substantiating the significance of LA, but the dramatic differences between the environments of the three sites are less often mentioned. Under such conditions, any superiority of nonlocal populations over local populations is likely to be swamped by the dramatically different climates among locations. Statistically speaking, the greater the differences among environments, the greater the expected effect of LA, which in statistical terms is the interaction between genotype and environment. When making inferences for restoration, the scale (variance) among test locations should match the ecological scale of the targeted restoration environments. Hereford (2009) suspected that estimates of the frequency of local superiority are biased upwards due the tendency of researchers to choose divergent environments for testing for the presence of LA, as a finding of local isn't best may be construed as a negative result. However, Leimu and Fischer (2008) tested for this source of bias and found no support for it.

In the Intermountain West USA, plant materials for rangeland use have been developed by three USDA agencies: the Forest Service, the Natural Resources Conservation Service (NRCS), and the Agricultural Research Service (ARS). Testing of rangeland plant materials is an ongoing process, having been conducted for decades by these three agencies. Increased resources are permitting an increase in test number, replication, and environmental diversity to better assess seedling establishment, plant cover, and persistence. For a given species, seeds for testing will be produced in common environments but will be spatially isolated to avoid confounding genetic effects with environmentally induced seed quality effects, a source of bias. These tests will permit hypothesis testing regarding (i) the relative adaptation of local versus nonlocal plant materials, (ii) the limits of adaptation of individual plant materials, and (iii) potential invasiveness. In the Intermountain West USA, evidence linking rangeland plantings to conservation benefits is lacking (Hardegree et al. 2011). Currently, this linkage can only be presumed through successful plant establishment (Hardegree et al. 2011); hence, data are needed to quantify the impact of various plant materials on ecosystem resilience and desired ecosystem services.

### The relative frequency of opportunity for nature versus restoration practice

Unfortunately, it is much easier for humanity to inflict damage on the landscape than to rectify it. The pristine expression of nature at a specific site is a transient product of unlikely-to-be-repeated sequences of fluctuating weather, climate, and biological immigration (Jackson et al. 1995). Opportunities to reverse degradation by seeding may depend on opportunistic and infrequent weather events, particularly for marginal environments (Neilson 1986; Harrington 1991). In restoration practice, there may be only a single opportunity to establish a successful seeding before invasive plants gain a foothold due to the so-called priority effect (Palmer et al. 1997). Thus, in such situations, a case can be made for restoration plant materials that display an increased likelihood of establishment success, given a single opportunity relative to local material.

## When local is or isn't best

### What are some situations in which it is likely that local is or isn't best?

1. When environmental conditions vary across sites, a genotype × environment interaction for fitness may permit natural selection for LA at individual sites (Kawecki and Ebert 2004), resulting in a LIB pattern. However, this selection may be confounded by gene flow and genetic drift, opposed by selection for temporal environmental variation, and constrained by a lack of genetic variation and genetic architecture of underlying traits (Kawecki and Ebert 2004) such that local is not necessarily best.
2. The classic examples of LIB are the tree species of the Pacific Northwest that are regarded as ‘specialists’, that is, those displaying little tolerance of seed transfer (Rehfeldt 1994; Johnson et al. 2004). In the Pacific Northwest, steep elevational gradients associated with adaptive variation to temperature and precipitation are present (Johnson et al. 2004). Such gradients or environmental heterogeneity across sites within a species' distribution increases the likelihood of finding that LIB (Jones 2013). Other ‘generalist’ species are relatively tolerant of seed transfer (Rehfeldt 1994; Johnson et al. 2004). In general, specialists evolve on sites that are constant, and generalists evolve on sites that are heterogeneous across space or time, but particularly across time (Kassen 2002). While generalists may evolve despite environmental heterogeneity across their distributions, they are more likely than specialists to occupy a distribution that is relatively homogeneous (Jones 2013). Many generalist species are widespread (Janzen 1985), and for such species, superior nonlocal populations may exist.
3. For populations on the edge of a species' distribution (Davis and Shaw 2001) or exposed to selection pressures atypical of the species as a whole, local is more likely to be best. However, performance of populations of species prone to inbreeding depression may be compromised by founder effects, inbreeding, or landscape fragmentation (Broadhurst et al. 2008; Weeks et al. 2011). Such genetically depauperate populations are more likely to display local maladaptation and are less likely to be best (Broadhurst et al. 2008; Leimu and Fischer 2008).
4. Local adaptation is conditioned in part by past climatic extremes, which may be infrequent. This ‘genetic memory’ may be recalled when extreme events recur (Montalvo et al. 1997), favoring a LIB pattern. However, genetic memory may be less relevant when environmental conditions have changed (Sgrò et al. 2011). Furthermore, if these novel conditions result in no-analog communities, that is, ones present nowhere else on Earth, it may be difficult to find pre-adapted material of a particular species anywhere (Williams and Jackson 2007).

‘Local is best’ is an assumption of the seed transfer zone model (Johnson et al. 2004), being based on the general idea that natural selection is an optimizing process. While the belief that LIB is widespread (McKay et al. 2005; Latta 2008), the overall evidence for LIB seems to be equivocal (Leimu and Fischer 2008; Hereford 2009). As Gould (1998) and Harper (1982) recognized, local can be expected to be reasonably well adapted under pristine conditions, although not necessarily ‘best’. However, adaptation is less likely when the environment has been modified. Consequently, rather than as a hard-and-fast rule, LIB may be better thought of as a context-dependent and testable assumption. However, when testing data are unavailable, a frequent occurrence, a local population may be the preferred option when seed is commercially available and when ecological conditions have not been modified to a great extent.
